# TRPC6-Mediated Zn^2+^ Influx Negatively Regulates Contractile Differentiation of Vascular Smooth Muscle Cells

**DOI:** 10.3390/biom15020267

**Published:** 2025-02-12

**Authors:** Chenlin Su, Xinya Mi, Tomoya Ito, Yuri Kato, Akiyuki Nishimura, Ryu Nagata, Yasuo Mori, Motohiro Nishida

**Affiliations:** 1 Graduate School of Pharmaceutical Sciences, Kyushu University, Fukuoka 812-8582, Japanyu-kato@phar.kyushu-u.ac.jp (Y.K.); 2 National Institute for Physiological Science (NIPS), National Institutes of Natural Sciences, Okazaki 444-8787, Japan; 3 Exploratory Research Center on Life and Living Systems (ExCELLS), National Institutes of Natural Science, Okazaki 444-8787, Japan; 4 Department of Physiological Sciences, SOKENDAI (School of Life Science, The Graduate University for Advanced Studies), Okazaki 444-8787, Japan; 5 Graduate School of Pharmaceutical Sciences, Osaka University, Osaka 565-0871, Japan; 6 Graduate School of Engineering, Kyoto University, Kyoto 615-8510, Japan

**Keywords:** Zn^2+^ influx, TRPC6 channel, phenotypic switching, vascular smooth muscle cells

## Abstract

Vascular smooth muscle cells (VSMCs) can dynamically change their phenotype between contractile and synthetic forms in response to environmental stress, which is pivotal in maintaining vascular homeostasis and mediating pathological remodeling of blood vessels. We previously reported that suppression of canonical transient receptor potential 6 (TRPC6) channel-mediated cation entry sustains VSMCs contractile phenotype and promotes the blood flow recovery after hindlimb ischemia in mice. We also reported that Zn^2+^, a metal biomolecule mobilized by TRPC6 channel activation, exerts potential beneficial effects on cardiac contractility and remodeling. Therefore, we hypothesized that TRPC6-mediated Zn^2+^ influx participates in phenotype switching of VSMCs and vascular remodeling. We established rat aortic smooth muscle cells (RAoSMCs) stably expressing wild type (WT) and Zn^2+^ only impermeable TRPC6 (KYD) mutant. Although the resting phenotypes were similar in both RAoSMCs, pharmacological TRPC6 activation by PPZ2 prevented the transforming growth factor (TGF) β-induced reduction in the intracellular Zn^2+^ amount and contractile differentiation in RAoSMCs (WT), but failed to prevent them in RAoSMCs (KYD). There were no significant differences in TRPC6-dependent cation currents among all RAoSMCs pretreated with or without TGFβ and/or PPZ2, suggesting that TRPC6 channels are functionally expressed in RAoSMCs regardless of their phenotype. Treatment of mice with PPZ2 attenuated the progression of vascular remodeling caused by chronic angiotensin II infusion. These results suggest that Zn^2+^ influx through TRPC6 channels negatively regulates the TGFβ-induced contractile differentiation of VSMCs and the progression of vascular remodeling in rodents.

## 1. Introduction

Mature arterial reconfiguration refers to structural and functional changes in the vessel wall that occur in response to pathological conditions, injury, or aging process [1,2]. Vascular smooth muscle cells (VSMCs), the major cell type located in the middle layer of the arterial vessel wall, contribute to the maintenance of vascular tone and structural stability by alternating with elastic fibers [3,4,5]. VSMCs exhibit significant plasticity, which allows for dynamic transitions between highly proliferative (synthetic) and fully differentiated (contractile) phenotypes, thereby ensuring vascular maturation [6]. Healthy mature VSMCs exhibit a limited synthetic ability [7], while in response to the pathogenesis of various human cardiovascular diseases, the contractile phenotype is converted into a dedifferentiated synthetic phenotype [8]. For example, in the pathological state of atherosclerosis, changes in the contractile phenotype of VSMCs lead to the narrowing of the vessel diameter and vascular stiffness [5,9,10,11,12,13,14]. On the other hand, with increased blood volume, vascular tone increases, which causes VSMCs to excessively transition to the contractile phenotype, resulting in chronic hypertension accompanied by a thickening of the medial layer [15]. Therefore, maintaining the ability of VSMCs to switch phenotypes is critical for sustaining vascular homeostasis.

Canonical subfamily members of transient receptor potential (TRPC) proteins form phospholipase C-linked receptor-operated Ca^2+^-permeable non-selective cation channels in vertebrates [16]. Among the TRPC1-TRPC7 subfamily, the pathophysiological roles of TRPC6 have been well investigated in VSMCs [17]. In cultured pulmonary arterial smooth muscle cells (PASMCs), TRPC6 expression is up-regulated after treatment with platelet-derived growth factor through the increase in store-operated Ca^2+^ entry, which contributes to hyperplasia by facilitating phenotype switching of PASMCs from contractile to synthetic [18,19]. We have reported that the increased TRPC6 channel activity negatively regulates the promotion of vessel maturation after hindlimb ischemia, potentially contributing to peripheral arterial disease [20]. Suppression of Ca^2+^ entry through the TRPC6 channel activated by ischemic stress could make it more switchable to contractile phenotype by reducing the interaction between TRPC6 and phosphatase and tensin homolog deleted on chromosome 10 (PTEN) [21]. However, TRPC6 does not only permeate Ca^2+^, Na^+^ and K^+^, but it also permeates other metal ions, such as Zn^2+^ and Fe^2＋.^. Of note, these characteristics are not conserved in most TRPC6-related TRPC3 and TRPC7 channels [22,23,24,25,26].

Zn^2+^ permeates from extracellular (approximately 10 nM) into the cells through transporters and ion channels, and most Zn^2+^ is believed to be tightly bound to intracellular organelles, for instance, enzymes and metallothioneins [27,28]. While Zn^2+^ binds with protein in a labile form, mobile intracellular Zn^2+^, approximately 100 pM, remains to function as a second messenger [27,29]. The pooled Zn^2+^ and mobile Zn^2+^ provide a different view of the physiological significance of Zn^2+^-binding protein interactions. The critical functions of cellular Zn^2+^ signaling underscore potential molecular pathways linking Zn^2+^ metabolism to disease progression [30]. The importance of Zn^2+^-permeable ion channels, especially in intracellular Zn^2+^ dynamics and Zn^2+^-mediated physiological and pathophysiological processes, is widely recognized [31]. Cellular Zn^2+^ capability can be estimated by the treatment with 2,2′-dithiodipyridine (DTDP), which oxidizes Zn^2+^-binding proteins and induces Zn^2+^ release from the intracellular Zn^2+^ pool [32]. Zn^2+^ participates in cell proliferation activity through several pathways [33]. For instance, Zn^2+^ is essential for insulin-like growth factor-I-induced cell proliferation [34] and nerve growth factor-induced differentiation [35].

Since several reports suggest the beneficial effects of Zn^2+^ in the cardiovascular system, we hypothesized that the Zn^2+^ influx mediated through the TRPC6 channel may contribute to the maintenance of vascular homeostasis by regulating VSMCs phenotype switching. To investigate the role of TRPC6-mediated Zn^2+^ influx in VSMCs phenotype switching, we established rat aortic smooth muscle cells (RAoSMCs) stably expressing TRPC6 wild-type (WT) and TRPC6 Zn^2+^-only impermeable mutant (KYD) [25]. We investigated whether pharmacological TRPC6 activation negatively regulates contractile differentiation of RAoSMCs by transforming growth factor (TGF) β stimulation. We also investigated whether pharmacological TRPC6 activation has a beneficial effect on vascular remodeling in angiotensin (Ang) II-infused mice.

## 2. Materials and Methods

### 2.1. Animals

All protocols using mice were reviewed and approved by the Animal Care and Use Committee at Kyushu University and performed according to Institutional Guidelines Concerning the Care and Handling of Experimental Animals (approval no. A24-167-1, A24-252-1).

We obtained 129/Sv male mice from the Comparative Medicine Branch, National Institute of Environmental Health Sciences (Research Triangle Park, NC, USA). All mice were group housed in individually ventilated cages (three or four animals per cage) with aspen wood chip bedding and kept under controlled environmental conditions (specific-pathogen-free area, 12 h light/dark cycle, room temperature 21–23 °C, and humidity 50–60%) with free access to standard laboratory food pellets (CLEA Rodent Diet CE-2, CLEA Japan, Tokyo, Japan) and water.

### 2.2. Vascular Remodeling Mice Model

Vascular remodeling was caused by continuous administration of Ang II (PEPTIDE, Cat# 4001, Osaka, Japan). A micro-osmotic pump (Alzet, Cat# 1002, Tokyo, Japan) filled with Ang II (2 mg/kg/day) with or without 2-[4-(2,3-dimethylphenyl)-piperazin-1-yl]-N-(2-ethoxyphenyl) acetamide (PPZ2, 2.5 mg/kg/day) [36], a TRPC3/6/7 channel activator, was embedded intraperitoneally into 129/Sv male mice (8–9 weeks old) for 14 consecutive days. Control mice were administered with the normal saline (NS). The pharmacological efficacy of Ang II was determined by the increase in blood pressure. Blood pressure was measured from the tail using a non-invasive tail-cuff system (Softron, Tokyo, Japan) while the mice were dressed in a special restraint suit and warmed with a heat mat (38 °C).

### 2.3. Immunocytochemistry

Thawed RAoSMCs were used for experiments after at least 5 passages, with almost all RAoSMCs showing a synthetic form, and discarded after 10 passages. RAoSMCs were cultured in culture medium (Dulbecco’s modified Eagle’s medium (DMEM, low glucose, Thermo Fisher Scientific, MA, USA) supplemented with 10% FBS, 1% penicillin and streptomycin and 2 μg/mL Puromycin) at 37 °C in a humidified atmosphere (5% CO_2_, 95% air) [25]. TRPC6 (WT)- and TRPC6 (KYD)-expressing RAoSMCs were established by using retrovirus, and positive cells were selected with puromycin [25]. TRPC6 (WT)- and TRPC6 (KYD)-expressing RAoSMCs were seeded at 3 × 10^3^ cells/200 μL on 4-well glass-bottom dishes. The cells were treated with 0.1% DMSO, 3 μM of PPZ2, 10 ng/mL of TGFβ with or without PPZ2 in 10 μM ZnCl_2_ supplied culture medium for 24 h, and then fixed with 4% paraformaldehyde in PBS (Fujifilm Wako, Cat# 045-29795, Osaka, Japan) for 15 min. After washing with PBS for 10 min three times, cells were blocked with blocking–permeabilization solution containing 0.2% Triton X-100 and 10% FBS in PBS for 1 h at room temperature. Cells were overnight incubated with the primary antibodies at 4 °C: anti-α-smooth muscle actin (α-SMA) (1:500 dilution; eBioscience, Cat# 14-9760-82, CA, USA), anti-smooth muscle 22 α (SM22α) (1:500 dilution; Abcam, Cat# 14106, Cambridge, UK) and anti-TRPC6 (1:300 dilution; Thermo Fisher Scientific, Cat# ACC-120, MA, USA). Alexa Fluor 488-conjugated anti-mouse IgG or 488-conjugated anti-rabbit IgG (1:500 dilution; Thermo Fisher Scientific, Cat# A11029 or Cat# A11034, MA, USA) secondary antibodies were applied for 2 h at room temperature in blocking–permeabilization solution. The nuclei were stained with 4′-6′-diamidino-2-phenylindole (DAPI, 1:10,000 dilution; DOJINDO, Cat# 340-7971, Kumamoto, Japan). The fluorescence images were acquired using a fluorescence microscope (BZ-X800; Keyence, Osaka, Japan) or a confocal microscope (LSM 900; ZEISS, Oberkochen, Germany), and the mean fluorescence intensity/pixel of each cell was analyzed with ImageJ2 (Version: 2.14.0/1.54f) software (National Institutes of Health, Bethesda, MD, USA).

### 2.4. Cell Proliferation Assay

Cell proliferation was assessed using a Cell Counting Kit (CCK)-8 (DOJINDO, Cat# 347-07621, Kumamoto, Japan). TRPC6 (WT)- and TRPC6 (KYD)-expressing RAoSMCs were seeded at 5 × 10^3^ cells in 100 μL each well on a 96-well plate. One day after plating, cells were treated with 0.1% DMSO or 3 μM of PPZ2 in 10 μM ZnCl2 supplied culture medium for 1 h. Subsequently, each group was co-treated with or without 10 ng/mL of TGFβ for 24 h. The value of wells with 0.1% DMSO was normalized as a control.

### 2.5. RAoSMCs Transfection

RAoSMCs were cultured in Dulbecco’s modified Eagle’s medium (DMEM, low glucose) supplemented with 10% FBS, 1% penicillin and streptomycin at 37 °C in a humidified atmosphere (5% CO_2_, 95% air). A fraction of RAoSMCs were plated in 35 mm culture dishes and transfected with plasmid DNAs (TRPC6 (WT) and TRPC6 (KYD) mutant) [37] using X-tremeGENE9 (Roche, Basel, Switzerland). One day after plating, cells were treated with 0.1% DMSO or 3 μM of PPZ2 in 10 μM ZnCl2 supplied culture medium for 1 h. Subsequently, each group was co-treated with or without 10 ng/mL of TGFβ for 24 h. Then, cells were reseeded onto glass coverslips (3 × 5 mm^2^) in 35 mm culture dishes for patch clamp.

### 2.6. Zn^2+^ Imaging

For the measurement of Zn^2+^ influx, TRPC6 (WT)- and TRPC6 (KYD)-expressing RAoSMCs were reseeded onto coverslips (3 × 5 mm^2^) in a 6-well plate with 2 mL culture medium. One day after plating, cells were treated with 0.1% DMSO or 3 μM of PPZ2 in 10 μM ZnCl2 supplied culture medium for 1 h. Subsequently, each group was co-treated with or without 10 ng/mL of TGFβ for 24 h. Then, cells were washed with 1× HBSS (10× HBSS, Thermo Fisher Scientific, Cat# 14065-056, MA, USA) and loaded with FluoZin-3 (2 μM) (Thermo Fisher Scientific, Cat# F24195, MA, USA) for 30 min at 37 °C [25]. After loading, the dye solution was replaced with 1× HBSS. Then, 50 μM of DTDP (Sigma, Cat# 2127-03-9, Darmastadt, Germany) was applied 2 min after starting measurement, and then N,N,Nʹ,Nʹ-tetrakis (2-pyridylmethyl)ethylenediamine (TPEN, 50 μM; Zn^2+^ chelator)(DOJINDO, Cat# 340-05411, Kumamoto, Japan) was applied 5 min after adding DTDP. Fluorescence images were recorded every 10 s and analyzed using a video image analysis system (Aquacosmos 2.6, Hamamatsu Photonics, Shizuoka, Japan).

### 2.7. Whole-Cell Patch-Clamp Techniques

The conventional whole-cell patch-clamp technique was used to record resting membrane potential and ion currents in the voltage-clamp models at room temperature with an EPC-10 patch-clamp amplifier (HEKA, Lambrecht, Germany). Patch electrodes with a resistance of 3–4 MΩ were made from G-1.5 mm borosilicate glass capillaries (Sutter Instrument, CA, USA). We have preliminarily confirmed using electrophysiological experiments that TRPC6 (KYD) mutant specifically lacks Zn^2+^ permeability compared with TRPC6 (WT) in HEK293 cells. In this study, the TRPC6 current [38] was measured by the whole-cell patch-clamp technique on TRPC6 WT and KYD mutant overexpressing RAoSMCs. Before patch-clamp measurement, RAoSMCs were treated with 0.1% DMSO or 3 μM of PPZ2 in 10 μM ZnCl2 supplied culture medium for 1 h. Subsequently, each group was co-treated with or without 10 ng/mL of TGFβ for 24 h. Cells were allowed to settle in the perfusion chamber in the external solution. TRPC6 was recorded in K^+^-free bathing solution including (in mM): 140 NaCl, 5.4 CsCl, 1 CaCl_2_, 1 MgCl_2_, 0.33 NaH_2_PO_4_, 5 HEPES and 5.5 glucose (pH 7.4, adjusted with NaOH). The pipette solution contained (in mM) 120 CsOH, 120 aspartate, 20 CsCl, 2 MgCl_2_, 5 EGTA, 1.5 CaCl_2_, 10 HEPES and 10 glucose (pH 7.2, adjusted with CsOH). Voltage ramps (−100 to +100 mV) of 250 ms were recorded every 2 s from a holding potential of −60 mV.

### 2.8. Immunohistochemical Analysis of Mouse Aortas

The aortas removed from mice were embedded in an optimal cutting temperature compound (Sakura Finetech, Tokyo, Japan) and frozen in liquid nitrogen. Frozen tissues were sliced at 10 μm slices by Leica CM1100 (Leica Biosystems, Nussloch, Germany). Sections were fixed with 4% paraformaldehyde for 15 min, then washed with PBS three times. Blocking of the sections was performed with a blocking–permeabilization solution containing 0.2% Triton X-100 and 10% FBS in PBS for 1 h at room temperature. Sections were stained overnight at 4 °C with primary anti-mouse α-SMA (1:500 dilution, eBioscience, Cat# 14-9760-82, CA, USA) and anti-mouse CD31 (1:200 dilution, BioLegend, Cat# 102401, CA, USA) antibodies, incubated for 2 h with secondary Alexa Fluor 488-conjugated anti-mouse IgG or Alexa Fluor 568-conjugated anti-rat IgG (1:500 dilution; Thermo Fisher Scientific, Cat# A11077, MA, USA) at room temperature in the blocking–permeabilization solution. The specimens were observed with a fluorescence microscope (BZ-X800; Keyence, Osaka, Japan). For the analysis of α-SMA and CD31 expression in aortas, fluorescence signal intensity within the region of interest (ROI) being made based on α-SMA fluorescence images was quantified, which was further normalized and represented as a fold increase from before chronic Ang II administration. ImageJ2 (Version: 2.14.0/1.54f) software (National Institutes of Health, Bethesda, MD, USA) was used for all the image analysis.

### 2.9. Statistical Analysis

All results are presented as the mean ± SEM from at least three independent experiments. Statistical analyses were performed using GraphPad Prism 9.0 (GraphPad Software, LaJolla, CA, USA), performing the Normality and Lognormality tests with the Shapiro–Wilk test for small sample sizes (N < 50) and the Kolmogorov–Smirnov test for larger sample sizes (N ≥ 50) [39]. When the test indicated *p* > 0.05, the data were considered normally distributed. At the same time, the graphic method Q-Q plot is used for the auxiliary test. When data were from three or more groups, one-way analysis of variance (ANOVA) with Tukey’s post hoc test was used for normally distributed data, and the Kruskal–Wallis test with the Dunn test was used for nonparametric data. Sample sizes were at least 5 animals per group subjected to statistical analysis. *p* values < 0.05 were considered statistically significant.

## 3. Results

### 3.1. Pharmacological Activation of TRPC6 Channel Prevents TGFβ-Induced Differentiation on TRPC6 (WT)-Expressing RAoSMCs

**Figure 1:**
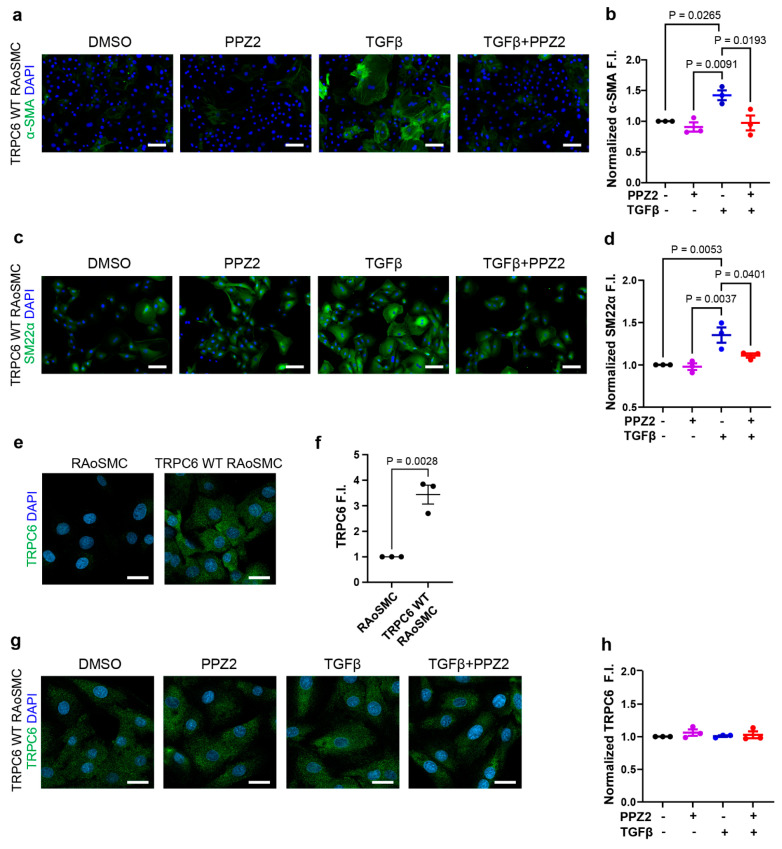
Pharmacological activation of TRPC6 prevents the TGFβ-induced contractile differentiation of TRPC6 (WT)-expressing RAoSMCs: (**a**,**b**) Representative images and quantification of α-SMA (green) immunofluorescence intensities in TRPC6 (WT)-expressing RAoSMCs. Scale bars = 50 μm. (**c**,**d**) Representative images and quantification of SM22α (green) immunofluorescence intensities in TRPC6 (WT)-expressing RAoSMCs. Scale bars = 50 μm. (**e**,**f**) Representative images and quantification of TRPC6 (green) immunofluorescence intensities in RAoSMCs and TRPC6 (WT)-expressing RAoSMCs. Scale bars = 30 μm. (**g**,**h**) Representative images and quantification of TRPC6 (green) immunofluorescence intensities in TRPC6 (WT)-expressing RAoSMCs. Scale bars = 30 μm. Nuclei were counterstained with DAPI (blue). Three separate experiments (N = 3) were averaged to produce the data. Data are shown as mean ± SEM. *p* < 0.05 using one-way ANOVA followed by Tukey’s post hoc test.

TGFβ is a key cytokine implicated in vascular remodeling, and it has been shown to drive VSMCs toward differentiation state [40,41,42,43,44]. We first examined if pharmacological activation of the TRPC6 channel negatively regulates the TGFβ-induced contractile differentiation of RAoSMCs. Activation of TRPC3/6/7 channels by PPZ2 treatment markedly decreased the fluorescence intensities of contractile markers, including α-SMA (Figure 1a,b) and SM22α (Figure 1c,d) in TGFβ-induced RAoSMCs. There were significant TRPC6 fluorescence intensities in TRPC6 (WT)-expressing RAoSMCs, compared with normal RAoSMCs (Figure 1e,f). The protein expression levels of TRPC6 (WT) were not affected by the treatment with PPZ2 or TGFβ (Figure 1g,h). As the expression level of TRPC6 in smooth muscle cells is much higher compared with that of TRPC3 and TRPC7 [45], these results suggest that activation of the TRPC6 channel negatively regulates the TGFβ-induced contractile differentiation of RAoSMCs.

### 3.2. Lacking Zn^2+^ Influx Activity of TRPC6 Fails to Prevent the TGFβ-Induced Differentiation of RAoSMCs by PPZ2 Treatment

**Figure 2:**
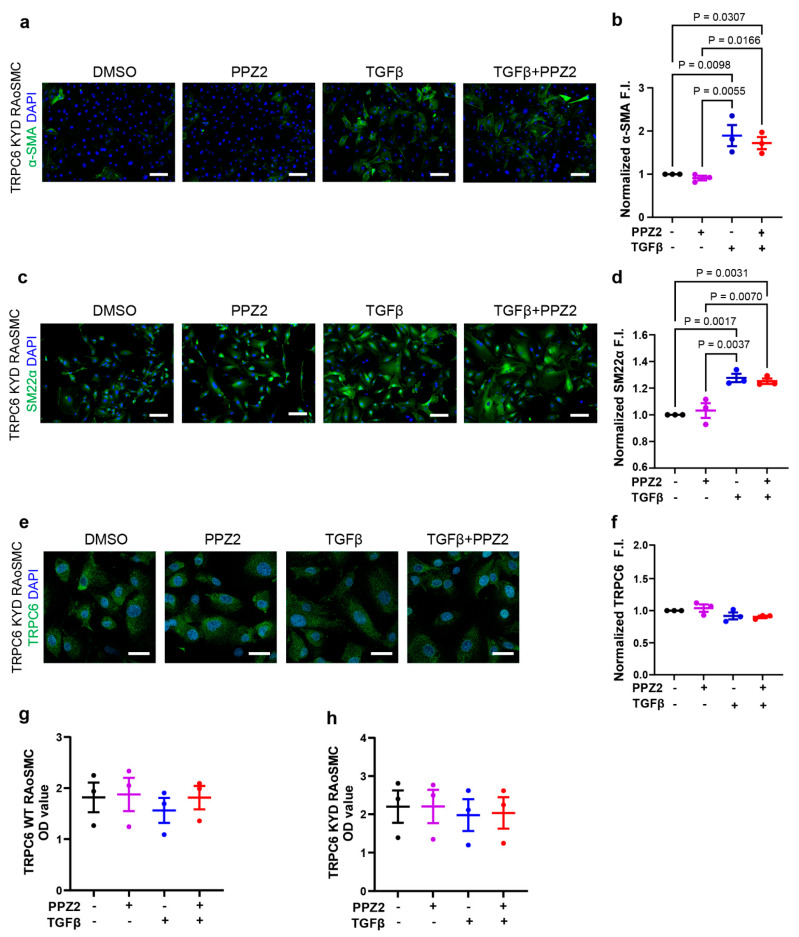
Requirement of TRPC6-mediated Zn^2+^ influx in PPZ2-induced prevention of TGFβ-induced contractile differentiation of RAoSMCs: (**a**,**b**) Representative images and quantification of α-SMA (green) immunofluorescence intensities in TRPC6 (KYD)-expressing RAoSMCs. Scale bars = 50 μm. (**c**,**d**) Representative images and quantification of SM22α (green) immunofluorescence intensities in TRPC6 (KYD)-expressing RAoSMCs. Scale bars = 50 μm. (**e**,**f**) Representative images and quantification of TRPC6 (green) immunofluorescence intensities in TRPC6 (KYD)-expressing RAoSMCs. Scale bars = 30 μm. Nuclei were counterstained with DAPI (blue). (**g**,**h**) Cell-proliferating activity was assessed by optical density (OD) values in TRPC6 (WT)- and TRPC6 (KYD)-expressing RAoSMCs. Three separate experiments (N = 3) were averaged to produce the data. Data are shown as mean ± SEM. *p* < 0.05 using one-way ANOVA followed by Tukey’s post hoc test.

To investigate whether TRPC6-mediated Zn^2+^ influx contributes to the prevention of TGFβ-induced VSMCs differentiation by PPZ2, we utilized Zn^2+^ impermeable TRPC6 mutant (TRPC6 (KYD))-expressing RAoSMCs, as previously reported [25]. The contractile markers of α-SMA (Figure 2a,b) and SM22α (Figure 2c,d) fluorescence intensities were similarly increased by TGFβ stimulation. However, the contractile marker intensity levels under treatment of PPZ2 were not reduced in TGFβ-stimulated RAoSMCs. The TRPC6 protein expression levels were also unaffected by treatment with PPZ2 or TGFβ (Figure 2e,f). The proliferating activities of TRPC6 (WT)- and TRPC6-(KYD)-expressing RAoSMCs were similar even though treated with PPZ2 and/or TGFβ (Figure 2g,h). These results indicate that TRPC6-mediated Zn^2+^ influx activity is critical for the prevention of TGFβ-induced VSMC differentiation by PPZ2.

### 3.3. Pharmacological Activation of TRPC6 by PPZ2 Reverses the TGFβ-Induced Reduction in Intracellular Zn^2+^ Amount in RAoSMCs

**Figure 3:**
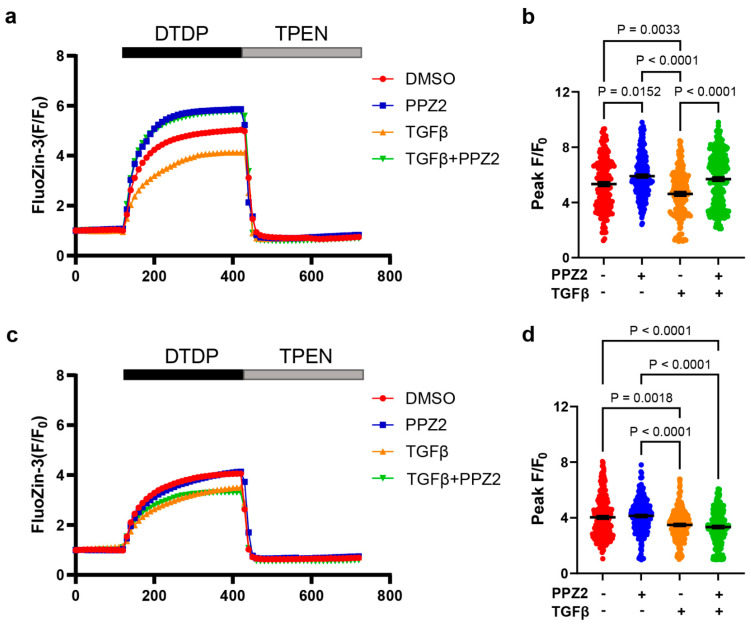
Activation of TRPC6-mediated Zn^2+^ influx reverses the reduction in intracellular Zn^2+^ pool in TGFβ-stimulated RAoSMCs: (**a**,**b**) Time courses and peak intensities of FluoZin-3 fluorescence evoked by DTDP in TRPC6 (WT)-expressing RAoSMCs. TPEN chelates intracellular free Zn^2+^. DMSO N = 184 cells; PPZ2 N = 242 cells; TGFβ N = 189 cells; TGFβ + PPZ2 N = 267 cells. (**c**,**d**) Time courses and peak intensities of FluoZin-3 fluorescence evoked by DTDP in TRPC6 (KYD)-expressing RAoSMCs. TPEN chelates intracellular free Zn^2+^. DMSO N = 267 cells; PPZ2 N = 265 cells; TGFβ N = 245 cells; TGFβ + PPZ2 N = 245 cells. Experiments were repeated at least 3 times. Data are shown as mean ± SEM. *p* < 0.05 using Kruskal–Wallis test with Dunn test.

Next, we investigated the relationship between intracellular Zn^2+^ amount and phenotype switching in VSMCs. The released amount of the pooled Zn^2+^ evoked by DTDP was significantly reduced in TGFβ-stimulated TRPC6 (WT)-expressing RAoSMCs, and this reduction was reversed by PPZ2 (Figure 3a,b). The released amount of the pooled Zn^2+^ evoked by DTDP was also reduced in TGFβ-induced TRPC6 (KYD)-expressing RAoSMCs, but this reduction was never reversed by PPZ2 (Figure 3c,d). These results suggest that PPZ2 contributes to the maintenance of an intracellular pooled Zn^2+^ amount in VSMCs through the TRPC6 channel.

### 3.4. Expression of Functional TRPC6 Protein Levels Are Not Changed in RAoSMCs (WT) and RAoSMCs (KYD) Regardless of Phenotype

**Figure 4:**
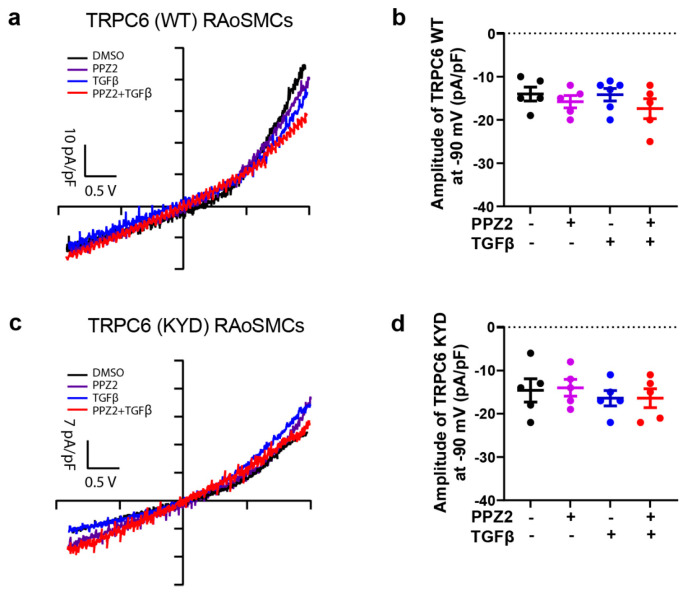
Similar electrophysiological properties of TRPC6-mediated currents in TRPC6 (WT)- and TRPC6 (KYD)-expressing RAoSMCs: (**a**,**c**) Representative leak-subtracted *I-V* relationships of TRPC6 currents recorded in TRPC6 (WT)- and TRPC6 (KYD)-expressing RAoSMCs pretreated with DMSO or PPZ2 (3 μM) and with or without TGFβ (10 ng/mL) for 24 h. N = 5 cells from 3 independent experiments, respectively. (**b**,**d**) The amplitude of TRPC6 current at −90 mV. Data are shown as mean ± SEM. *p* < 0.05 using one-way ANOVA followed by Tukey’s post hoc test.

We further investigated whether phenotype switching or PPZ2 treatment has some impact on the expression of functional TRPC6 channels in RAoSMCs. TRPC6 channel can permeate cations such as Na^+^, Ca^2+^ and K^+^. The total TRPC6-mediated currents detected by whole-cell patch clamp recording were not changed by 24 h PPZ2 or TGFβ pretreatment in TRPC6 (WT)-expressing RAoSMCs (Figure 4a). No significant differences were observed in TRPC6-mediated current amplitude at −90 mV (Figure 4b). Of note, the electrophysiological properties and currents amplitude of TRPC6 remained unchanged in TRPC6 (KYD)-expressing RAoSMCs, and PPZ2 and/or TGFβ treatment had no impact on TRPC6-mediated currents at −90 mV (Figure 4c,d). These results clearly suggest that the expression levels of functional TRPC6 channels are not changed among all experiments.

### 3.5. PPZ2 Attenuates the Ang II-Induced Vascular Remodeling in Mouse Aorta

**Figure 5:**
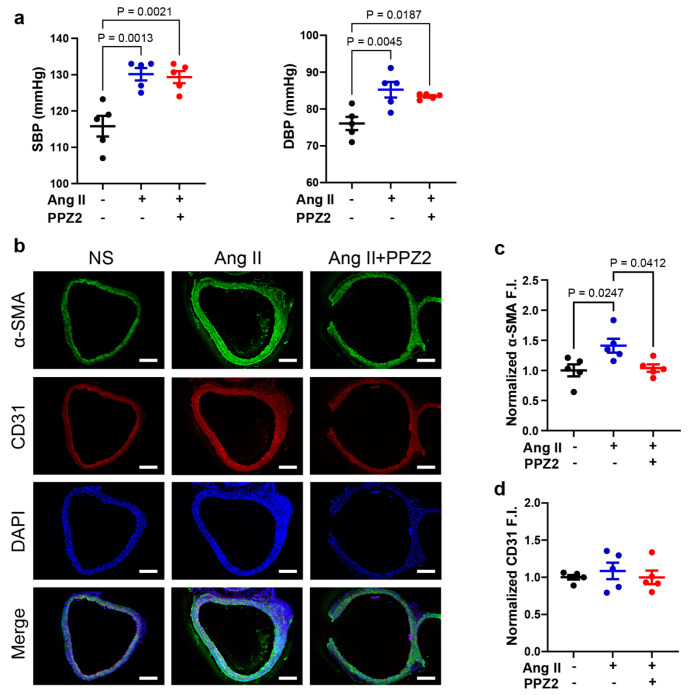
PPZ2 attenuates Ang II-induced vascular remodeling in mice aorta: (**a**) Systolic blood pressure (SBP) and diastolic blood pressure (DBP) in mice. (**b**) Representative images of α-SMA (green) and CD31 (red) immunofluorescence staining in mice aortas. (**c**) Quantification of α-SMA immunofluorescence intensities. (**d**) Quantification of CD31 immunofluorescence intensities. Nuclei were counterstained with DAPI (blue). Scale bars = 200 µm. Five mice were tested in each group (N = 5). Data are shown as mean ± SEM. *p* < 0.05 using one-way ANOVA followed by Tukey’s post hoc test.

Ang II enhances the expression or function of TGFβ receptors on VSMCs to modulate cell growth [46]. We finally examined whether PPZ2 treatment attenuates vascular remodeling caused by Ang II in mice. We confirmed that Ang II-treated mice caused severe hypertension to the same extent in both DMSO- (systolic blood pressure, 130.1 ± 1.7 mmHg) and PPZ2-treated mice (systolic blood pressure, 129.3 ± 1.7 mmHg) (Figure 5a). However, the fluorescence intensities of α-SMA were increased in the media layer of the arterial vessel wall in chronic Ang II-treated mice, while this increase was canceled by PPZ2 treatment (Figure 5b,c). This result suggests that activation of the TRPC6 channel attenuates pathological vascular remodeling in mice partly through the Zn^2+^ influx-mediated signaling pathway.

## 4. Discussion

Our findings suggest the pivotal role of TRPC6-mediated Zn^2+^ influx in regulating VSMCs phenotypic switching, with broader implications in vascular remodeling. Using RAoSMCs (KYD), we successfully separate the role of TRPC6-mediated Zn^2+^ influx from TRPC6-mediated other cation influxes and revealed that pharmacological activation of TRPC6 by PPZ2 negatively regulates TGFβ-induced contractile differentiation of RAoSMCs through Zn^2+^ mobilization. Based on this in vitro observation, we also demonstrated using Ang II-treated mice that PPZ2 treatment attenuated pathological vascular remodeling. These findings offer new insights into vascular plasticity and remodeling mechanisms and significant implications for cardiovascular disease management.

**Figure 6:**
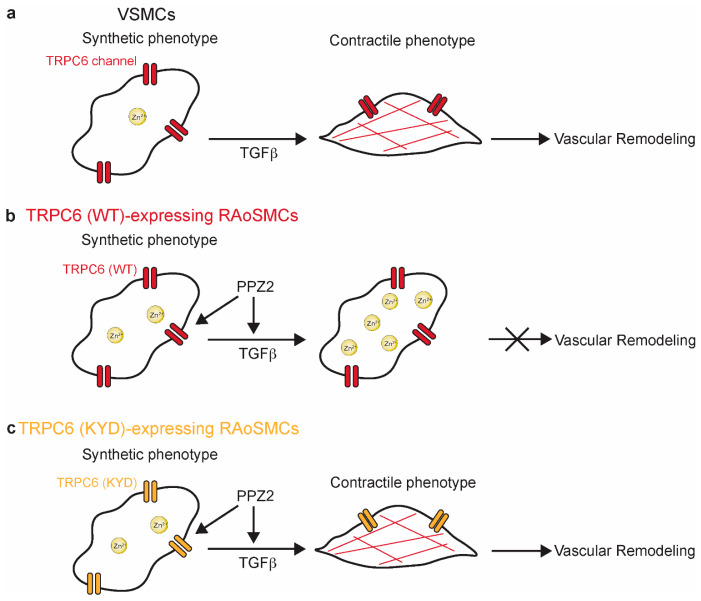
Schema of the role of TRPC6-mediated Zn^2+^ influx in negative regulation of TGFβ-induced VSMC contractile differentiation and vascular remodeling: (**a**) Synthetic phenotype of VSMCs switches into contractile phenotype under TGFβ stimulation, which further induced vascular remodeling in vivo. (**b**,**c**) Activation of TRPC6 channel by PPZ2 negatively regulates contractile dedifferentiation in TRPC6 (WT)-expressing RAoSMCs (**b**), which was failed by the specific inhibition of Zn^2+^ permeability on TRPC6 (**c**).

Compared to other TRPC channels, the specific Zn^2+^ permeability of the TRPC6 channel introduces a new dimension to its regulatory function in cellular homeostasis. Previous studies predominantly focused on TRPC6-mediated Ca^2+^ influx in vascular pathologies. TRPC6 deficiency impairs the maintenance of the contractile phenotype in arterial VSMCs, as evidenced by reduced expression of contractile markers, enhanced VSMCs proliferation, and migration, as well as exacerbated neointimal hyperplasia and luminal stenosis in the common carotid arteries (CCA) of TRPC6^(−/−)^ mice [47]. We previously reported that activation of the TRPC6 channel by diacylglycerol induces Ca^2+^ entry, promoting synthetic switching of VSMCs through membrane depolarization and Ca^2+^-dependent interaction with PTEN [21]. PTEN represses downstream PI3K/Akt signaling, which is necessary for contractile differentiation, by dephosphorylating phosphatidylinositol-3, 4, 5-trisphosphate. In contrast, mobile Zn^2+^ is reported to inhibit PTEN, leading to activating the PI3K/Akt pathway of interleukin-2 signaling in T-cells, human airway epithelium, and neurodegeneration [48]. Otherwise, Zn^2+^ deficiency reduces the proliferation of transplanted tumors in host animals [49] and decreases the proliferation of rat aorta-origin VSMCs (A7r5 VSMCs) and aortic VSMCs under non-calcifying and calcifying conditions [50]. As the electrophysiological TRPC6 (KYD) channel activity was equivalent to that of TRPC6 (WT) channel activity in RAoSMCs, the mechanism of Zn^2+^-mediated suppression of TGFβ-induced contractile differentiation must be distinct from Ca^2+^-dependent regulation (Figure 6). Further investigation is required to elucidate how TRPC6-mediated Zn^2+^ influx negatively regulates the contractile differentiation, including the underlying signaling pathways.

Our findings highlight Zn^2+^ as a critical second messenger in TRPC6-mediated regulation of VSMCs. However, the causal relationship between Zn^2+^ dynamics and vascular plasticity has been obscure. Therefore, beyond Ca^2+^ signaling, we utilized DTDP to release the intracellular zinc pool to evaluate the interaction of Zn^2+^ and phenotype regulation in VSMCs. Our data suggested that pharmacological activation of TRPC6 by PPZ2 alleviated the TGFβ-induced contractile differentiation and reduction in intracellular Zn^2+^ amount in RAoSMCs (WT) but not in RAoSMCs (KYD). These differences suggest that TRPC6-mediated Zn^2+^ influx negatively regulates TGFβ-induced VSMC differentiation through maintaining intracellular Zn^2+^ homeostasis.

Vascular injury, characterized by endothelial dysfunction, structural remodeling, inflammation and fibrosis, significantly contributes to the progression of cardiovascular diseases. Cellular processes underlying vascular injury include imbalanced VSMC plasticity, which results in maladaptive phenotypic switching [6]. Ang II signaling pathways become activated with age and contribute to developing arteriosclerosis and vascular senescence. Ang II is one of the numerous factors implicated in vascular injury, it can be converted into smaller, functionally active peptides and play a role in regulating vascular tone and structure [51]. The chronic infusion of Ang II induces the development of several cardiovascular diseases, including cardiac fibrosis [52], atherosclerosis [53] and aortic aneurysms in mice [54]. Although our Ang II infusion model was insufficient to cause vascular injury, we could observe vascular remodeling after a 4-week Ang II infusion. We demonstrated that activation of the TRPC6 channel by PPZ2 attenuated the Ang II-induced vascular remodeling in mice. Further analyses focusing on TRPC6-mediated Zn^2+^ influx in vivo should be necessary to establish the therapeutic potential of TRPC6 channel activation against pathological vascular remodeling.

## 5. Conclusions

Our findings highlight the pivotal role of TRPC6-mediated Zn^2+^ influx in modulating VSMC phenotypic plasticity and vascular remodeling, providing novel insights into its potential as a therapeutic target for cardiovascular diseases. These results not only expand the understanding of Zn^2+^ signaling in vascular biology but also underscore the clinical significance of TRPC6 in regulating vascular function. Future investigations should focus on elucidating the molecular mechanisms underlying TRPC6-mediated Zn^2+^ signaling, exploring its role across various vascular cell types to fully realize its therapeutic potential.

## Data Availability

All data generated or analyzed during this study are included in this published article and are available from the corresponding author upon reasonable request.
